# Investigation of genetic markers associated to type 2 diabetes mellitus in Santarém-Pará

**DOI:** 10.1590/1678-4685-GMB-2023-0107

**Published:** 2024-08-05

**Authors:** Adjanny Estela Santos de Souza, Caio Henrique Silva da Silva, Rita de Cássia Silva de Oliveira, Ana Paula Araújo Guimarães, Aylla Núbia Lima Martins da Silva, Isabela Guerreiro Diniz, Haiala Soter Silva de Oliveira, Diego Sarmento de Sousa, Fernanda Andreza de Pinho Lott Figueiredo, Greice de Lemos Cardoso Costa, João Farias Guerreiro

**Affiliations:** 1 Universidade do Estado do Pará, Faculdade de Medicina, Departamento de Morfologia e Ciências Fisiológicas, Belém, PA, Brazil.; 2 Universidade Federal do Pará, Instituto de Ciências Biológicas, Laboratório de Genética Humana e Médica, Belém, PA, Brazil.; 3 Universidade Federal do Oeste do Pará (UFOPA), Santarém, PA, Brazil.; 4 Centro Universitário da Amazônia (UNAMA), Santarém, PA, Brazil.

**Keywords:** Genetic polymorphism, population genetics, diabetes mellitus, single nucleotides polymorphisms

## Abstract

Genetic, epigenetic and environmental factors play an important role in the genesis of Type 2 Diabetes Mellitus (T2D). In the genetic context, one of the strategies used to investigate possible associations with diabetes is the search for Single Nucleotide Polymorphisms (SNPs), involving the comparison of alelle frequencies, the phenotypic variations and other relevant factors, such as environmental influences and lifestyle choices, Thus, the aim of this study was to find the relationship of risk variants for T2D in SNPs (*rs4994*) in the *ADRB3* gene; (*rs1799854*) in the *ABCC8* gene; (*rs7901695* and *rs12255372*) in the *TCF7L2* gene; and (*rs8050136*) in the *FTO* gene in a sample of the population of the municipality of Santarém (PA), Brazilian Amazon, in the northern region of Brazil. *ABCC8* (*rs1799854* C>T) showed a statistically significant association with T2D. Each chosen gene and SNP has been previously implicated in T2D risk according to existing scientific literature, owing to their roles in glucose regulation and body fat.

## Introduction

Diabetes Mellitus (DM) is a metabolic disease characterized by persistent hyperglycemia resulting from defects in insulin secretion by pancreatic beta cells or decreased sensitivity to insulin by other cells in the body (Lyra et al., 2006). With regard to the etiopathogenesis of diabetes, the disease can be classified as type 1 diabetes (T1D), type 2 diabetes (T2D), gestational diabetes (GDM), and other types of diabetes (SBD, 2022). T2D is responsible for 90-95% of diabetes cases. Furthermore, due to its complexity, this disease represents one of the biggest challenges for Primary Health Care with regard to prevention and treatment, which aims to prevent systemic complications related to the underlying inflammatory process in individuals with the disease (Leal et al., 2017; Romanciuc, 2017; Manoel et al., 2021).

The etiology of T2DM is mainly related to excessive consumption of carbohydrates and fats, excessive weight, sedentary behavior, a family history of diabetes, and insulin resistance. Thus, the disease depends on environmental and genetic factors. Among the risk factors for the development of the disease, some are highlighted, such as overweight (BMI>25) and central obesity, hypertriglyceridemia, hypertension, HDL < 40 mg/dL, age over 45 years, and family history (Tavares et al., 2010). The influence of age, although not fully understood, has an intrinsic relationship with environmental and genetic factors. With regard to environmental factors, it is worth stressing that a change in lifestyle to a more sedentary behavior, reinforced by metabolic changes in old age, makes the group of older people more vulnerable to the development of T2DM (Malafaia and Buglia, 2019).

Genetic, epigenetic, and environmental factors play an important role in the development of the disease. As for genetic factors, Genome-Wide Association studies (GWAs) have identified T2D susceptible in various populations, these studies have shown over 400 genetic risk variants at 250 loci for T2D (Carlson et al., 2013; Dziewulska et al., 2018). Other studies have also shown the contribution of genetic factors to the development of T2D in families with diabetic individuals, with concordance of 70% for monozygotic twins and only 20-30% for dizygotic twins (Newman et al., 1987; Kaprio et al., 1992). Another important observation is that the risk of T2D is about 40% when one of the parents is affected and 70% when both are affected (Köbberling and Tillil, 1982; Groop et al., 1996).

In this genetic context, one of the strategies used to investigate possible genetic associations with diabetes is the research of Single Nucleotide Polymorphisms (SNPs). As observed in some studies, the presence of SNPs *rs1799854* in the ATP Binding Cassette Subfamily C Member 8 (*ABCC8*) gene; *rs4994* in the β3-Adrenergic Receptors (*ADRB3*) gene; and *rs8050136* in the Fat Mass And Obesity Associated/Alpha-Ketoglutarate Dependent Dioxygenase (*FTO*) gene have a direct association with obesity, one of the risk factors for T2D, as it is one of the main causes of insulin-related disorders (Horikoshi et al., 2007; Cruz et al., 2010; Diniz et al., 2022). Furthermore, the study by Guzmán et al. (2010) shows that overexpression of the Transcription Factor 7-like 2 (*TCF7L2*) gene may be related to decreased insulin secretion, which leads to hyperglycemia.

Santarém is a city located in the west of the state of Pará, at the confluence of the Tapajós and Amazon rivers. It is one of the oldest cities in the Brazilian Amazon, and has a strong history of miscegenation, since it was founded and colonized by Portuguese explorers, but also has a significant presence of African and Indigenous people. This contributes to the genetic risk factors, because there is a higher likelihood of inheriting a combination of genetic variants associated with diabetes. In addition to this, the unique lifestyle and dietary habits prevalent in this population increase the risk of diabetes (Santos, 2004; Sousa *et al.*, 2020).

In this sense, this study aims to investigate the association of risk variants in SNPs (*rs4994*) in the *ADRB3* gene; (*1799854*) in the *ABCC8* gene; (*rs7901695* and *rs12255372*) in the *TCF7L2* gene, and (*rs8050136*) in the *FTO* gene with type 2 diabetes (T2D) in a sample of the population of Santarém, in the state of Pará, in the North Region of Brazil, known as the Amazon Region, where the rates of T2D are high. This research aims to improve our understanding on the molecular mechanisms underlying chronic hyperglycemia and to identify individuals at risk of developing the disease early on. These findings could lead to effective hygiene-dietary interventions or medical treatments, potentially preventing and reversing the metabolic state of T2D in these diverse populations.

## Subjects and Methods

### Type of research

This is a descriptive cross-sectional epidemiological research with a quantitative approach, consisting of a case-control study (Pereira, 1995; Fontelles, 2010). The project was submitted to the Ethics Committee in Research of the João de Barros Barreto University Hospital, according to the approval term of protocol no. 2137/2010.

The study was conducted in Santarém-Pará-Brazil with a total of 410 individuals. Of these, 209 patients had T2D and 201 were controls (individuals with age equal to or greater than 40 years, without symptoms and/or previous diabetes diagnosis, without first-degree relatives with diabetes). Diabetic patients were registered in Hiperdia, a program for the care of hypertensive and diabetic patients within the Unified Health System (SUS), and were using medications provided by SUS (Metformin 500 mg; Simvastatin 20 mg and 40 mg). For this reason, the clinical data of these patients may show variations due to glycemic and cholesterol control and may be underestimated when compared to diabetic patients without treatment.

A sociodemographic interview was conducted with the participants using a questionnaire. Anthropometric evaluation was then carried out using Body Mass Index (BMI) and waist circumference (WC). Blood pressure (BP) was measured using a digital monitor and participants were then instructed to collect blood samples for biochemical and genetic analysis.

After a 12-hour fast, blood samples (5 mL) were collected from the patients through venipuncture. The concentration of triglycerides (TG), total cholesterol (TC), high-density lipoproteins (HDL-cholesterol) and glucose were determined using an enzymatic-colorimetric method, according to the manufacturer’s instructions. Hemoglobin A1c (HbA1c) was measured using the modified Trivelli microchromatographic-colorimetric method and low-density lipoprotein (LDL-cholesterol) was calculated using the Friedewald formula. 

### Genotyping of Single Nucleotide Polymorphisms (SNPs)

About 1 mL of peripheral blood was obtained from each patient and each control individual for genomic DNA extraction, using the conventional phenol-chloroform extraction method (Old and Higgs, 1993) with some modifications.

The polymorphisms of the *ADRB3* (*rs4994*), *ABCC8* (*rs1799854*), *FTO* (*rs8050136*), and *TCF7L2* (*rs7901695* and *rs12255372*) genes were identified using Real-Time Polymerase Chain Reaction (RT-PCR), with a commercial assay developed by Applied BioSystems - TaqMan^®^, with adaptations in the genotyping standard protocol. Genotyping was done using a TaqMan SNP genotyping assay (Applied BioSystems, Foster City, CA, USA) according to the manufacturer’s instructions. Pre-designed probes were ordered for genotyping analysis. Approximately 10-50 ng of DNA was amplified with 5 μl of 2X TaqMan Universal PCR master mix, 0.5 μl of 40X primer and TaqMan probe mix. The cycles were 10 min at 95 °C, followed by 40 cycles of 15 s at 92 °C and 1 min at 60 °C. Allelic discrimination was performed on an Applied Biosystems RT-PCR system - Realtime (PCR). The genes were selected for this study based on previous studies that have shown the association of variants with obesity and/or type 2 diabetes in continental populations.

### Statistical analyses

For comparison between continuous biological variables between the T2D and control groups, a Student’s t-test was performed for variables with a normal distribution, Mann-Whitney’s U-test for variables that did not have a normal distribution (p<0.05), and Pearson’s χ2 test for categorical variables. For the comparison between the two groups (control and T2D), the Bonferroni correction was applied. Logistic regression analysis was used to verify possible associations between the SNPs investigated and T2D, under a priori genotypic models (dominant, codominant, and recessive) performed to calculate specific allele risk probabilities. The odds ratio (OR) was calculated with a 95% confidence interval to estimate the relative risk and strength of association, with an OR above 1 associated with an increased chance of a given characteristic occurring and an OR less than 1 the opposite. The p-values, after adjusted for sex and age, were calculated. These analyses were performed using the Statistical Package for the Social Sciences (SPSS) for Windows, version 20.0 (SPSS Inc., Chicago, IL, USA).

## Results

### Characteristics of the study sample

The biochemical and anthropometric characteristics of patients with T2D and of the healthy control group are presented in Table 1. As expected, individuals with T2D, compared to the control group, had significantly higher values for BMI, glucose, and triglycerides (p=0.000), while exhibiting lower levels of HDL-cholesterol (p=0.001).

**Table 1-:** Clinical characteristics of the studied population in Santarém/Pará.

Characteristics	Diabetes (209) (Minimum-maximum)	Control (201) (Minimum-maximum))	P
AGE	62.33 ± 10.7(33-86)	61.40 ± 12.5(36-97)	0.256
Gender %(N) F/M	76.1%(159)/23.9%(50)	68.7%(138)/31.3%(63)	0.093
BMI	27.03± 4.1(17.45-37.48)	26.25 ± 4.93(17.12-49.31)	0.032
Glycemia	200.54 ± 93.2(59-530)	87.46 ± 8.0(65-107)	0.000
SBP	145.16 ± 24.3(70-258)	143.18 ± 23.8(90- 232)	0.364
DBP	90.32 ± 15.8(45-160)	91.56 ± 14.8(50- 132)	0.195
Triglycerides	255.11 ± 180.2(55-375)	188.75 ± 130.8(30- 750)	0.000
Total cholesterol	196.85 ± 56.0(73-375)	194.77 ± 43.7(76-338)	0.936
HDL	41.36±9.6	45.43±11.7	0.001
LDL	110.15±46.1	113.37±40.4	0.392
Classification by weight			
Normal weight	33%	42.8%	0.101
Overweight	44%	39.8%	
Obese	23%	17.4%	
Fasting blood glucose			
Normal	7.7%	99.5%	0.000
Altered	15.3%	0.5%	
Elevated	77%	0.0	
Blood pressure			
Normal	27.3%	35.8%	0.062
Elevated	72.7%	64.2%	
Triglyceride			
Desirable	45%	67.7%	0.000
Elevated	55%	32.3%	
Cholesterol			
Desirable	81.3%	87.6%	0.083
Increased	18.7%	12.4%	

MI: Body mass index; SBP: Systolic blood pressure; DBP: Dyastolic blood pressure.

### Polymorphisms in *ABCC8*, *ADRB3*, *FTO*, and *TCF7L2* genes

The observed allelic frequencies for the five genetic variants in the genes investigated in the present study and related to T2D in the population of Santarém, along with the frequencies found in continental populations of Africans, Americans, Europeans, Southeast Asians, and Eastern Asians (“1000 Genomes Project”) (Table 2). For the *rs1799854* C>T variant in the *ABCC8* gene, it was observed that the mutant allele (allele T) had a high allelic frequency, above 40%, similar the allelic frequency observed in European, American, and East Asian populations. The highest allelic frequency for the *ABCC8 rs1799854* C>T variant was observed in the American population at 53.6% (allele T). The American subset of the “1000 Genomes Project” is composed of mixed populations from Puerto Rico, Colombia, and Mexico.

**Table 2-:** Allelic frequencies of the 5 single nucleotide polymorphisms (SNPs) related to diabetes in Amazon/PA population and in continental populations from the 1000 Genomes Project (%).

			Populations					
Gene/SNP	Allele	SANTARÉM	AFR	AMR	EUR	EAS	SAS	ALL
*ABCC8* *rs1799854*	C	0.505	0.862	0.464	0.580	0.449	0.681	0.632
	T	0.495	0.138	0.536	0.420	0.551	0.319	0.368
*ADRB3* *rs4994*	T	0.830	0.905	0.880	0.918	0.869	0.843	0.885
	C	0.169	0.095	0.120	0.082	0.131	0.157	0.115
*FTO* *rs8050136*	C	0.710	0.567	0.745	0.586	0.834	0.711	0.678
	A	0.286	0.433	0.255	0.414	0.166	0.289	0.322
*TCF7L2* *rs7901695*	T	0.721	0.564	0.744	0.656	0.977	0.704	0.718
	C	0.278	0.436	0.256	0.344	0.023	0.296	0.282
*TCF7L2* *rs12255372*	G	0.801	0.698	0.781	0.708	0.990	0.779	0.786
	T	0.198	0.302	0.219	0.292	0.010	0.221	0.214

Santarém-Amazônia/Pará; EUR, European; AFR, African; AMR, American; EAS, East Asian; SAS, Southeast Asian.

### Genotype-phenotype association

*ABCC8* (*rs1799854* C>T) showed a statistically significant association with T2D. A difference in the distribution pattern of the TT genotype frequencies was observed between individuals with T2D and controls (28.7% vs 23.9% [P= 0.042]). Furthermore, allele T significantly increased the risk for T2D compared to allele C OR=1.34 95% CI 1.02-1.76 P= 0.036. Significant associations were also observed for the co-dominant models (P=0.042 and 0.034, CC vs CT, TT) and dominant (P= 0.029, CC vs CT+TT) models, but not for the recessive model (P= 0.251. CC+CT vs TT) (Table 3). For the other variants *ADRB3* (*rs4994*), *FTO* (*rs 8050136*), TCFL7 (*rs7901695* and *rs12255372*-G>T), no statistically significant differences were observed.

**Table 3-:** Comparison of the adjusted odds ratio for three genetic models for genetic polymorphisms related to diabetes in the population of Santarém/PA.

Gene (rsId)	Genotype/all	Case N(209)	Control N(201)	Allelic OR (95% IC)	Co-dominant OR (95% IC)	Dominant OR (95% IC)	Recessive OR (95% IC)
Gene *ABCC8* *rs1799854*				C vs T	CC vs CT. TT	CC vs CT+TT	CC+CT vs TT
	CC	47 (22.5%)	65(32.3%)	1.34 (1.02-1.76)	1.0	1.0	1.0
	CT	102(48.8)	88(43.8%)	P=0.036	1.65(1.01-2.68)	1.65(1.05-0.55)	1.28(0.83-2.02)
	TT	60(28.7%)	48(23.9%)		P=0.042	P=0.029	P=0.251
	ALLELE C	196(46.9)	218(54.2%)		1.80(1.04-3.12)		
	ALLELET	222(53.1)	184(45.8%)		P=0.034		
Gene *ADRB3* *rs4994*				T vs C	TT vs TC. CC	TT vs TC+CC	TT+TC vs CC
	TT	145(69.4%)	144(71.6%)	0.88(0.61-1.24)	1.0	1.0	1.0
	TC	53(25.4%)	50(24.9%)	P=0.440	0.99(0.62-1.57)	1.06(0.69-0.64)	1.58(0.59-4.26)
	CC	11(5.3%)	7(3.5%)		P=0.980	P=0.775	P=0.359
	ALLELE T	343(82.1%)	338(84.1%)		1.48(0.54-4.03)		
	ALLELE C	75(17.9%)	64(15.9%)		P=0.439		
Gene *FTO* *rs8050136*				C vs A	CC vs CA. AA	CC vs CA+AA	CC+CA vs AA
	CC	106(50.7%)	106(52.7%)	0.92(0.68-1.25)	1.0	1.0	1.0
	CA	83(39.7%)	78(38.8%)	P=0.620	1.41(0.74-1.73)	1.11(0.75-1.64)	1.49(0.57-2.27)
	AA	20(9.6%)	17(8.5%)		P=0.540	P=0.591	P=0.692
	ALLELE C	295(70.6%)	290(72.1)%		1.12(0.54-2.30)		
	ALLELE A	123(29.4%)	112(27.9%)		P=0.749		
Gene *TCF7L2* *rs7901695*				T vs C	TT vs TC. CC	TT vs TC+CC	TT+TC vs CC
	TT	110(52.6%)	110(54.7%)	0.93(0.68-1.26)	1.0	1.0	1.0
	TC	79(37.8%)	73(36.3%)	P=0.665	1.04(0.68-1.26)	1.07(0.66-1.71)	1.09(0.53-2.15)
	CC	20(9.6%)	18(9.0%)		P>0.05	P=0.778	P=0.809
	ALLELE T	299(71.5%)	293(72.9%)		1.13(0.49-2.61		
	ALLELE C	119(28.5%)	109(27.1%)		P=0.760		
Gene TCFL2 *rs12255372*				G vs T	GG vs GT. TT	GG vs GT+TT	GG+CT vs TT
	GG	138(66.0%)	134(66.7%)	0.97(0.69-1.37)	1	1	1
	GT	58(27.8%)	55(27.4%)	P=0.873	1.01(0.63-1.61)	0.99(0.60-1.64)	0.98(0.40-4.37)
	TT	13(6.2%)	12(6.0%)		P=0.946	P=0.989	P=0.971
	ALLELE G	334(79.9%)	323(80.3%)		1.04(0.41-2.60)		
	ALLELE T	84(20.1%)	79(19.7%)		P=0.929		

P < 0.05 and OR with corresponding 95% CI > 1 are represented in bold. Odds ratios (OR) and corresponding 95% confidence interval (CI) adjusted for age, sex, and BMI as variables.

## Discussion

In this study, the association of SNPs *ADRB3* (*rs4994*), *ABCC8* (*rs1799854*), *FTO* (*rs8050136*), and *TCF7L2* (*rs7901695* and *rs12255372*) with T2D was investigated in an admixed Amazonian population from the interior of the North Region of the state of Pará, in the city of Santarém. The research of polymorphisms associated with T2D had never been investigated in this population, making this the first study of its kind.

Among the 5 Brazilian regions, the North Region leads in the prevalence of obesity cases, especially due to poor nutrition and sedentary lifestyle (Malveira et al., 2021), a condition that contributes significantly to the development of T2D diabetes through disorders in insulin metabolism. In this study, weight did not express a statistically significant value (p=0.101), but if we observe the BMI, we will see a statistically significant difference (p=0.032), a variable that can be influenced by sex hormones in metabolism (Satler et al., 2021).

In this sample, it was possible to identify the presence of biochemical and anthropometric characteristics typical of T2D, showing metabolic changes due to the disease, which may be related to unbalanced diets and the social and economic conditions of the sample (Malveira et al., 2021). Elevated levels of triglycerides, blood glucose, BMI, and low levels of HDL-cholesterol were observed, and the prevalence of hypertriglyceridemia was higher in the T2D patient group (55%) compared to the estimated rate for adult Brazilians (31.2%) (Schmidt et al., 2015). Hypertriglyceridemia is the most common lipid disorder in patients with diabetes and is associated with an increased risk of cardiovascular disease (Hokanson and Austin, 1996; Miller et al., 1998; Rosenson et al., 2002). Additionally, there were elevated mean values of systolic and diastolic blood pressure in the study population, showing a considerable number of hypertensive individuals. The coexistence of hypertension and T2D double the risks of cardiovascular events in diabetic individuals, when compared to non-diabetic individuals (Curb, 1996; Penalva, 2008; Cryer et al., 2016).

### Allelic frequencies in the Santarém population

Genetic variants associated with diabetes and obesity phenotypes were predominantly demonstrated in European and Asian populations (Wang et al., 2009); however, such allelic variants have also shown distribution in populations from other continents, such as the American population (Carlson et al., 2013).

Compared to the allelic frequencies of the populations of the “1000 Genomes Project,” the data obtained showed similarity in the distribution of the allelic frequencies of the polymorphisms investigated in studies of continental populations with the samples of this study. Therefore, from the four genes investigated in this study, the *rs1799854* variant in the *ABCC8* gene, related to T2D (Wang et al., 2009), had the highest frequency (49.5%) of the risk allele (T). It is worth mentioning that the T allele of this variant also had a high distribution in the American and Asian populations at 53.6% and 55.1%, respectively. Diniz et al. (2022) also showed high frequencies of this same variant in indigenous populations of the state of Pará (53.3%). The similarity between these results denotes the importance of investigating this genetic variant as a susceptibility factor for T2D in mixed and native populations.

The gene frequency in the Santarém sample can be explained by the mixed character of its population, predominantly represented by Portuguese, Africans, and indigenous people (Santos, 2004), whose proportions were estimated as 39%, 28%, 33%, respectively (Santos *et al.*, 1996). For this reason, it is possible to point out that the contribution of the genetic risk factor has a great relevance in these populations and the result of the impact of these factors can be compared with other studies carried out with American and European populations (Rodrigues, 2018).

### Genotype-phenotype relationship

This study found an association between the risk allele (T) and T2D only for variant *rs1799854* in the *ABCC8* gene. This gene encodes the sulfonylurea 1 receptor protein, which participates along with K+ channels, expressed by pancreatic beta cells, in the regulation and secretion of insulin in response to glucose at beta cells.

The intronic polymorphism *rs1799854*, as also observed in other studies, is associated with hyperglycemia observed in the population with T2D, in addition to abdominal obesity, body fat and high BMI, characteristics also observed in a study performed with indigenous populations of the Brazilian Amazon and in a Polish study (Pietrzak-Nowacka et al., 2012; Rodríguez-Rivera et al., 2019; Diniz et al., 2022). The same variant was also associated with T2D in Japanese (Sakamoto et al., 2007), Caucasian (Florez et al., 2004) and Chinese populations (Zhou et al., 2009).

The present study showed that the T allele and CT and TT genotypes, in the dominant model of the *rs1799854* variant in the *ABCC8* gene, were significantly associated with the risk of developing T2D. This association observed in the group of patients with T2D has also been demonstrated in other studies (Meirhaeghe et al., 2001; Niu et al., 2005; Yokoi et al., 2006; Gonen et al., 2012). 

On the other hand, some studies about the influence of SNP *rs1799854* did not demonstrate association between this variant and T2D susceptibility in Asian and Caucasian populations (Lv et al., 2011; Venkatesan et al., 2014). However, the effect of this genetic variant on specific, unidentified subgroups with T2D cannot be excluded. Additionally, a number of environmental, genetic and statistical factors may be subject to variations in the results observed in these different populations. 

Other variants were studied such as *ADRB3* (*rs4994*), *FTO* (*rs8050136*), and *TCF7L2* (*rs7901695* and *rs12255372*). Collins et al. (1994) e Yamakita et al. (2010) showed that decreased expression of *ADRB3* in adipose tissue may contribute to the obesity phenotype with insulin resistance; and Diniz et al. (2022) found statistically significant results that demonstrate this relation in native populations of the Amazon. Studies show that some *FTO* gene polymorphisms are related with obesity in different ethnic groups, such as Caucasians (Hunt et al., 2008), and asians (Chang et al., 2008; Hotta et al., 2008; Yajnik et al., 2009). As for the *TCFL72* variants, studies show that it is a strong marker associated with T2D and have been robustly reported by GWA studies and consistently replicated in multiple populations of different genetic origins.

However, these variants have not shown statistically significant correlation with T2D in the population of this study. Nevertheless, this does not completely excludes the association between these variants and the increase in BMI and body fat (factors that are directly connected with obesity and T2D) in Santarém population.

The findings in this study may suggest the influence of different epigenetic and environmental factors in different population groups. In order to clarify the multi-factorial contributions related to T2D in mixed populations of the Brazilian Amazon, additional studies of gene-gene and gene-environment interactions are necessary (Bosque-Plata et al., 2021).

## Conclusion

This study investigated the association of SNPs *ADRB3* (*rs4994*), *ABCC8* (*rs1799854*), *FTO* (*rs8050136*) and *TCF7L2* (*rs7901695* and *rs12255372*) with type 2 diabetes in a sample of the Amazonian population. It was observed that the T allele and CT and TT genotypes, in the dominant model of the *rs1799854* variant in the *ABCC8* gene, were significantly associated with the risk for developing type 2 diabetes and could be considered a good genetic marker in studies related to type 2 diabetes, both in admixed populations of the Brazilian Amazon and in native populations. Therefore, it is crucial that further studies be conducted to arrive at this conclusion more robustly.

The combination of multiple genetic and environmental factors contributes to the pathogenesis of type 2 diabetes (T2D), so the association between this polymorphism and T2D can be used as a risk marker for the disease and its complications. However, the precise mechanism of development and progression is not well understood. There is a need for further studies to identify individuals with T2D carrying these variants, as well as to understand the mechanisms by which these polymorphisms affect metabolic characteristics associated with the disease, as well as the gene-environment interaction in the predisposition to T2D, contributing to the elucidation of the potential biological role in the pathogenesis of T2D.

Knowledge of the individual genetic predisposition profile for type 2 diabetes (T2D) and associated comorbidities may contribute to the effective prevention of T2D and its complications, through the use of differentiated prevention and control strategies according to the needs of each group, considering the profile of patients through self-care orientation programs, physical activity, nutritional guidance, monitoring of glycemic control and lipid profile in Health Units, contributing to the reduction of morbidity and mortality related to diabetes, as well as the costs of treating the disease and its complications.
